# Association Between Changes in Timing of Spring Onset and Asthma Hospitalization in Maryland

**DOI:** 10.1001/jamanetworkopen.2020.7551

**Published:** 2020-07-06

**Authors:** Amir Sapkota, Yan Dong, Linze Li, Ghassem Asrar, Yuyu Zhou, Xuecao Li, Frances Coates, Adam J. Spanier, Jonathan Matz, Leonard Bielory, Allison G. Breitenother, Clifford Mitchell, Chengsheng Jiang

**Affiliations:** 1 Maryland Institute for Applied Environmental Health, University of Maryland School of Public Health, College Park; 2 School of Remote Sensing and Information Engineering, Wuhan University, Wuhan, Hubei, China; 3 Joint Global Change Research Institute, Pacific Northwest National Laboratory, College Park, Maryland; 4 Department of Geological and Atmospheric Sciences, Iowa State University, Ames; 5 Aerobiology Research Laboratories, Nepean, Ontario, Canada; 6 University of Maryland School of Medicine, Baltimore; 7 Chesapeake Clinical Research, Chesapeake, Maryland; 8 Department of Medicine, Hackensack Meridian School of Medicine at Seton Hall University, Nutley, New Jersey; 9 Center for Environmental Prediction, Rutgers University, New Brunswick, New Jersey; 10 Environmental Health Bureau, Maryland Department of Health, Baltimore

## Abstract

**Importance:**

Ongoing climate change is affecting the health of communities across the globe. While direct consequences, including morbidity and mortality tied to increases in the frequency of extreme weather events, have received significant attention, indirect health effects, particularly those associated with climate change–driven disruptions in ecosystems, are less understood.

**Objective:**

To investigate how ongoing changes in the timing of spring onset related to climate change are associated with rates of asthma hospitalization in Maryland.

**Design, Setting, and Participants:**

This cross-sectional study of 29 257 patients with asthma used general additive (quasi Poisson) and mixed-effect (negative binomial) models to investigate the association between changes in the timing of spring onset, detected using satellite observations, and the risk of asthma hospitalization in Maryland from 2001 to 2012. Data analysis was conducted from January 2016 to March 2019.

**Exposures:**

Phenology data, derived from the National Aeronautics and Space Administration’s Moderate Resolution Imaging Spectroradiometer, were used to calculate location-specific median dates for start of season from 2001 to 2012. How the start of season for a given year and location deviated from the long-term average was calculated and categorized as very early, early, normal, or late.

**Main Outcomes and Measures:**

Daily asthma hospitalization in Maryland during the spring season (ie, March to May).

**Results:**

There were 108 358 total asthma hospitalizations during the study period, of which 29 257 (27.0%; 14 379 [49.1%] non-Hispanic black patients; 17 877 [61.1%] women) took place during springtime. In the unadjusted model, very early (incident rate ratio [IRR], 1.17; 95% CI, 1.07-1.28) and late (IRR, 1.07; 95% CI, 1.00-1.15) onset of spring were associated with increased risk of asthma hospitalization. When the analysis was adjusted for extreme heat events and concentrations of particulate matter with an aerodynamic diameter less than 2.5 μm, the risk remained significant for very early spring onset (IRR, 1.10; 95% CI, 1.02-1.20) but not for late spring onset (IRR, 1.03; 95% CI, 0.97-1.11).

**Conclusions and Relevance:**

These results suggest that ongoing changes in the timing of spring onset, which are related to climate variability and change, are associated with asthma hospitalization. Given the high burden of allergic diseases and the number of individuals sensitized to tree pollen, these findings serve as a wake-up call to public health and medical communities regarding the need to anticipate and adapt to the ongoing changes in the timing and severity of the spring allergy season.

## Key Points

**Question:**

Are changes in timing of spring onset related to ongoing climate variability and change associated with rates of asthma hospitalization during the spring season?

**Findings:**

In this cross-sectional study of 29 257 patients with asthma, very early onset of spring was associated with a 17% increase in hospital admissions for asthma and late onset of spring was associated with a 7% increase.

**Meaning:**

In this study, the observed associations between changes in timing of spring onset and asthma hospitalizations were likely associated with pollen dynamics, ie, early onset of spring could increase the length of the tree pollen season while late onset may increase pollen concentrations because of simultaneous blooming.

## Teaser

This cross-sectional study investigates how ongoing changes in the timing of spring onset related to climate change are associated with rates of asthma hospitalization in Maryland.

## Introduction

Previous studies have linked attributes of climate change, such as increased frequency of extreme heat, drought, hurricanes, flooding, and wildfires, with both chronic and acute health outcomes.^1,2^ However, there is a paucity of data regarding how climate-induced ecological changes, such as vegetation phenology and the timing of spring onset, may affect the burden of allergic diseases.

Phenological events, such as dormancy, bud formation, and flowering among temperate deciduous trees, are related to environmental cues, including photoperiod and temperature.^3,4^ While the photoperiod at a given location remains the same, the temperature cue has undergone considerable change during the past several decades in response to climate change.^3^ Using historical records of in situ observations, previous studies have shown that such increases in temperature are associated with earlier onset of spring events, including bud burst, leaf-out, and flowering, across the northern hemisphere.^5,6,7,8,9,10,11,12^ These studies suggest that flowering dates have advanced by 2 to 10 days per 1-°C increase in mean temperature,^5,6,7^ with more pronounced changes observed among species that bloom earlier in the spring.^13,14^ Others have used Earth system observations to link increasing temperature with earlier onset of start of spring (SOS) in large geographic areas covered with temperate deciduous forest.^15,16,17^ While the methods of studies that used historical records of in situ observations made at the individual plant-species level are distinctly different from those based on remote sensing observations of ecosystems or regions, both sets of studies clearly point to a direct association between increasing temperature and earlier SOS in the northern hemisphere. Others have suggested such phenological changes to be the most sensitive indicator of ecological response to climate change.^6,18,19,20,21^

Springtime phenological events, such as bud burst and flowering, are tied to the timing of production and release of tree pollen, an important source and trigger of springtime allergies.^22,23^ Thus, ongoing changes in springtime phenology attributed to climate variability and change can affect exposure dynamics for allergenic tree pollen.^22,23,24,25,26^ This is significant because allergenic pollen is among the leading risk factors that is known to worsen asthma symptoms.^27,28,29,30,31,32,33^ Currently more than 26 million US residents live with asthma,^34,35^ of whom approximately 50% reported having asthma attacks in the past year.^36^ The burden of asthma on the health care system is substantial, accounting for an estimated 10.6 million physician visits and more than 400 000 discharges from inpatient care, costing the US economy approximately $56 billion per year in total costs.^36,37,38,39^

A number of studies have hypothesized changes in pollen exposure dynamics as the critical link between climate change–related alterations in springtime flowering phenology and allergic disease burden, including asthma.^22,26,40,41,42^ However, to our knowledge, no studies to date have provided quantitative data supporting an end-to-end association between climate change–driven alterations in plant phenology, pollen concentration, and asthma hospitalization. Here we provide empirical evidence supporting an association between changes in timing of SOS and asthma hospitalization (2001-2012) using remote sensing–based plant phenology data and springtime daily hospitalization data.

## Methods

### Hospitalization Data

Inpatient hospital admission data for asthma (*International Classification of Diseases, Ninth Revision* [*ICD*-*9*] principal diagnosis code, 493) were obtained from the Maryland Department of Health. The hospitalization data covered January 2001 through December 2012 for the entire state of Maryland. Our main objective was to investigate how ongoing changes in the timing of SOS are associated with the asthma burden in Maryland, potentially by altering exposure dynamics for tree pollen during spring. Therefore, we only considered springtime hospitalization data (ie, March to May) with an assumption that asthma hospitalization during the summer, fall, or winter seasons would not be affected by tree pollen exposure in the spring. The hospitalization data included county of residence, age, sex, race/ethnicity, and date of hospitalization.^43^ This study followed the Strengthening the Reporting of Observational Studies in Epidemiology (STROBE) reporting guideline. The institutional review boards at the University of Maryland, College Park, and the Maryland Department of Health approved the use of data. The requirement for informed consent was waived because the study involved deidentified information only.

### Phenology Data

We downloaded the phenology product, derived using the National Aeronautics and Space Administration’s Moderate Resolution Imaging Spectroradiometer observations, from USGS.^44^ The phenology product we used was derived from time series data of normalized difference vegetation index (NDVI), as described previously.^45,46^ Product developers excluded poor-quality observations, including cloud contaminated pixels, and applied smoothing functions to improve the raw NDVI time series data.^46^ We designated SOS when the NDVI time series data showed a notable increase in positive slope in spring.^45^ Because the spring season in Maryland comprises March to May, SOS values outside this range (ie, June to February) were excluded. Likewise, deciduous trees are the primary source of tree pollen in Maryland during spring. Thus, we used the National Land Cover Database 2006 with 250 m spatial resolution^47^ to exclude areas covered with evergreen forest, mixed forest, shrub, and grassland. This process can mitigate the effect of the heterogeneity of land cover types on SOS. We used all pixel values within a county boundary covered with deciduous forest to calculate median SOS values for a given year. We then used these annual SOS values for the period from 2001 to 2010 to calculate a 10-year median SOS value (ie, day of year) for each county in Maryland. Finally, we calculated yearly deviation from the median value by subtracting yearly SOS for each county (2001-2012) from their respective 10-year median value.

Because speciated pollen data for Maryland were not available during the study period, we used pollen monitoring data from eastern Canada, provided by Aerobiology Research Laboratories, to evaluate the association between timing of spring onset in Eastern Canada, determined using satellite observations, and tree pollen dynamics in the same area. To achieve this, we used pollen data (2001-2012) from 5 monitoring stations located in the Canadian cities of Kingston (44° N), Ottawa (45° N), Montreal (45° N), Sherbrooke (45° N), and Quebec (47° N) and determined the length of the pollen season for birch, oak, and poplar pollen, as described previously.^23^ We then compared these pollen season lengths across SOS deviations observed at the same location as the pollen monitor. We chose northeast Canada because of the availability of speciated pollen data collected by investigators at Aerobiology Research Laboratories during the past 20 years and the abundance of deciduous forest, which is also present in Maryland.

### Statistical Analysis

We investigated the association between deviation in SOS and asthma hospitalization in Maryland using 2 types of models. First, we used a general additive model (quasi-Poisson), which included deviation in SOS as a continuous predictor to characterize the shape of the exposure response curve. We then used a mixed-effect model (negative binomial), which included deviation in SOS as a categorical variable, as follows: very early (ie, >10 days early), early (3-10 days early), normal (3 days early to 3 days late), and late (>3 days late). In both cases, we performed univariate and multivariate analysis, with the multivariate models adjusted for seasonal concentration of particulate matter with an aerodynamic diameter less than 2.5 μm, number of extreme heat events, and county-level poverty rate, based on the 2012 American Community Survey. We also stratified the analysis by age group, race/ethnicity, and urban/rural status. All analyses were performed at the county level. All statistical analyses were conducted using R statistical software version 3.5.0 (R Project for Statistical Computing). Statistical significance was set at *P* < .05.

## Results

Of 108 358 overall asthma hospitalizations in Maryland from 2001 to 2012, 29 257 (27.0%) occurred during springtime (Table 1). Most patients were women (17 877 [61.1%]), lived in urban areas (25 833 [88.3%]), and were non-Hispanic black (14 379 [49.1%]) or non-Hispanic white (12 151 [41.5%]) individuals. The springtime hospitalization pattern was similar to the overall hospitalization pattern across demographic characteristics, including age, race/ethnicity, sex, and urbanization (Table 1). The actual rates were slightly higher during the spring season, particularly among patients aged 4 years or younger (37.9 per 10 000 overall vs 40.9 per 10 000 during spring) and 65 years or older (24.1 per 10 000 overall vs 27.7 per 10 000 during spring). We observed considerable variability in the timing of SOS across the 24 counties (25 days early to 3 day late) in a given year as well as overall variability across the 12-year period (25 days early to 11 days late) (eFigure in the Supplement).

**Table 1.** Demographic Characteristics of the Study Population

Characteristic	Total population, No. (%) (N = 5 785 496)	Asthma hospitalizations during 12-y period			
		Total (n = 108 358)		Springtime (n = 29 257)	
		No. (%)	Rate per 10 000	No. (%)	Rate per 10 000
Age, y					
0-4	365 258 (6.3)	16 620 (15.3)	37.9	4478 (15.3)	40.9
5-17	985 445 (17.0)	15 274 (14.1)	12.9	4363 (14.9)	14.8
18-64	3 719 067 (64.3)	55 725 (51.4)	12.5	14 466 (49.4)	13.0
≥65	715 726 (12.4)	20 738 (19.1)	24.1	5949 (20.3)	27.7
Race/ethnicity					
Hispanic	472 285 (8.2)	2933 (2.7)	5.2	845 (2.9)	6.0
Non-Hispanic					
Black	1 675 532 (29.0)	54 635 (50.4)	27.2	14 379 (49.1)	28.6
White	3 163 295 (54.7)	43 910 (40.5)	11.6	12 151 (41.5)	12.8
Other	474 384 (8.2)	3328 (3.1)	5.8	921 (3.1)	6.5
Unknown	NA	3552 (3.3)	NA	961 (3.3)	NA
Sex					
Women	2 986 621 (51.6)	66 295 (61.2)	18.5	17 877 (61.1)	20.0
Men	2 798 875 (48.4)	42 062 (38.8)	12.5	11 379 (38.9)	13.6
Urbanization					
Rural	658 321 (11.4)	12 668 (11.7)	16.0	3424 (11.7)	17.3
Urban	5 127 175 (88.6)	95 690 (88.3)	15.6	25 833 (88.3)	16.8

Abbreviation: NA, not applicable.

**Figure 1.**
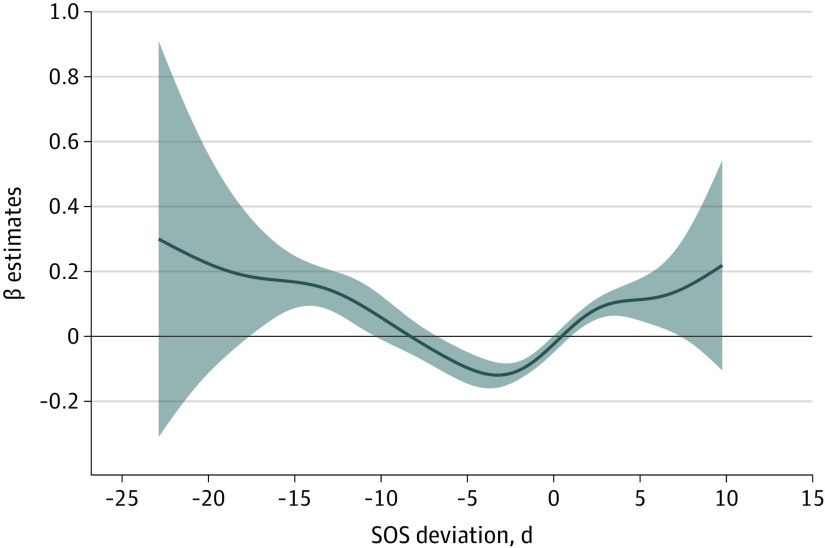
Regression Coefficient for Association of Deviation in Start of Spring (SOS) With Risk of Asthma Hospitalization in Maryland, 2001 to 2012 Shaded areas represent 95% CIs.

The overall association between changes in timing of SOS and risk of asthma hospitalization in Maryland was obtained using a generalized additive model (Figure 1). Very early and late onset of SOS was associated with an increased risk of springtime asthma hospitalization, with a noted dip (protective effect) in the exposure response function for SOS deviation of −6 to 0 days. Figure 1 also shows that the overall deviation in SOS ranged from −25 to 11 days, meaning the absolute magnitude of negative SOS deviation (ie, early onset of spring) was higher (ie, 25 days early) compared with the positive SOS deviation (ie, 11 days late). We further analyzed the data using a mixed-effect model, in which deviation in SOS was divided into 4 categories (ie, very early, early, normal, and late). In the unadjusted model, both very early (incident rate ratio [IRR], 1.17; 95% CI, 1.07-1.28) and late (IRR, 1.07; 95% CI, 1.00-1.15) SOS were associated with an increased risk of asthma hospitalization (Table 2). When the analysis was adjusted for extreme heat events and concentration of particular matter with a diameter less than 2.5 μm during the spring season (model 2), the risk of asthma hospitalization remained significant for very early SOS (IRR, 1.10; 95% CI, 1.02-1.20); however, late SOS was no longer associated with risk (IRR, 1.03; 95% CI, 0.97-1.11) (Table 2). Further adjustment for poverty level in model 3 did not change our results (Table 2).

**Table 2.** IRRs for Deviation in Start of Spring and Asthma Hospitalization in Maryland During 2001 to 2012

Covariate	IRR (95% CI)		
	Model 1^a^	Model 2^b^	Model 3^c^
Phenology			
Normal	1 [Reference]	1 [Reference]	1 [Reference]
Very early	1.17 (1.07-1.28)	1.10 (1.02-1.20)	1.10 (1.01-1.20)
Early	0.96 (0.89-1.03)	0.95 (0.88-1.03)	0.95 (0.88-1.04)
Late	1.07 (1.00-1.15)	1.03 (0.97-1.11)	1.03 (0.97-1.11)
Extreme heat event	NA	1.00 (0.99-1.01)	1.00 (0.99-1.01)
PM_2.5_ concentration	NA	0.94 (0.87-1.03)	0.94 (0.87-1.02)
Poverty	NA	NA	1.05 (1.02-1.08)

Abbreviations: IRR, incidence rate ratio; NA, not applicable; PM_2.5_, particulate matter with an aerodynamic diameter less than 2.5 μm.

^a^ Model 1 was unadjusted.

^b^ Model 2 was adjusted for extreme heat event and PM_2.5_ concentration.

^c^ Model 3 was additionally adjusted for poverty.

**Figure 2.**
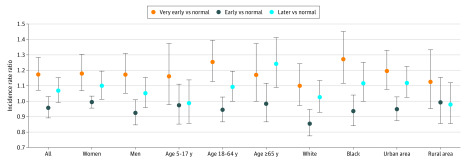
Stratified Analysis for Changes in Timing of Spring Onset and Risk of Asthma Hospitalization in Maryland, 2001 to 2012

We stratified the analysis to investigate whether the association between changes in the timing of SOS and asthma hospitalization varied across sex, age group (ie, 5-17, 18-64, and ≥65 years), race (ie, white and black), and urban and rural areas (Figure 2). Across all categories, very early onset of SOS was associated with increased risk of asthma hospitalization. The risk associated with the late onset of SOS was less consistent, with significantly increased risk observed among women (IRR, 1.09; 95% CI, 1.02-1.17), those aged 18 to 64 years (IRR, 1.10; 95% CI, 1.02-1.19), those aged 65 years and older (IRR, 1.22; 95% CI, 1.09-1.41), black individuals (IRR, 1.13; 95% CI, 1.03-1.24), and urban populations (IRR, 1.14; 95% CI, 1.05-1.22) (Figure 2).

**Figure 3.**
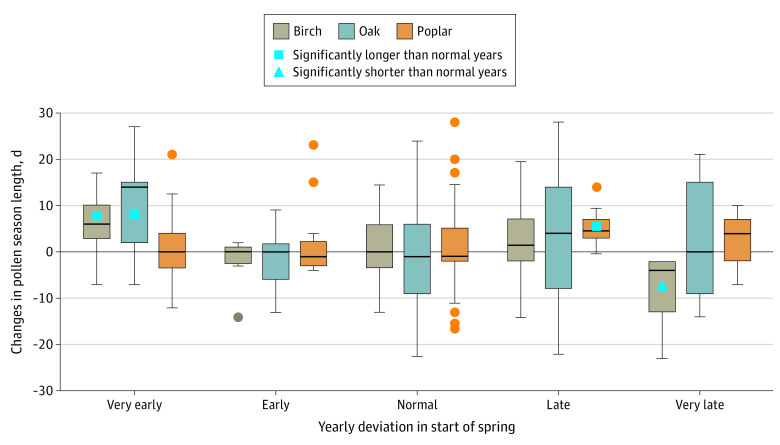
Changes in Pollen Season Length Across Categories of Deviation in Start of Spring The center line represents the median value, with upper and lower edges of the box representing the 75th and 25th percentile value, respectively. The whiskers represent the highest and the lowest values, excluding the outliers, which are indicated by dots.

We observed that very early SOS in eastern Canada was associated with increased pollen season length for birch and oak pollen (birch: mean [SD] difference, 4.6 [7.1] days; *P* = .01; oak: mean [SD] difference, 11.6 [12.9] days, *P* = .001) in the same area (Figure 3). Likewise, very late SOS was associated with shortened pollen season length for birch (mean [SD] difference, 9.1 [7.8] d; *P* < .001) but not for oak.

## Discussion

Previous studies have suggested that plant phenology, such as timing of SOS, may be the most sensitive indicator of ecological response to climate variability and change.^6,18,19,48^ Here, we showed that very early spring onset is associated with increased risk of asthma hospitalization in Maryland. This risk was consistent across age, race, and urban and rural categories. We also observed a higher risk of asthma hospitalization with late onset of spring, although only among selected subgroups (women, adults, black individuals, and urban residents).

A potential explanation for this association is that changes in the timing of spring onset alter tree pollen dynamics, including start, end, and length of pollen season, as well as the timing and intensity of peak pollen concentration during the spring season. Changes in timing of SOS are associated with changes in tree pollen dynamics, including timing of start and peak pollen concentration and pollen season length. These findings led us to hypothesize that the increased risk of springtime asthma hospitalization associated with changes in timing of SOS is mediated through tree pollen dynamics, ie, early spring onset lengthens tree pollen exposure while late spring onset may increase peak pollen concentration because different species of trees bloom simultaneously in response to delayed SOS. Under this scenario, the duration of the pollen season may be short, but the actual ambient concentration can be significantly higher because of simultaneous bloom. Future studies using asthma hospitalization and extensive pollen monitoring data from the same area are needed to confirm this mechanistic pathway.

Most previous studies investigating the association between climate change and impaired health have focused on direct as well as indirect health effects related to the increased frequency of extreme heat and precipitation events, changes in air quality and vector distribution, and foodborne and waterborne illnesses.^1,2^ Studies suggesting a direct link between climate change and the burden of allergic diseases have done so based on 3 different sets of thematic observational studies that have linked increased CO_2_ concentrations with higher pollen production, warmer air and surface temperatures with early SOS and longer pollen seasons, and higher pollen exposures with increased risk of asthma hospitalizations.^5,6,7,15,16,17,28,29,49,50,51^ While these are highly plausible hypotheses, to our knowledge, our study is the first that closes this loop by providing empirical evidence associating changes in the timing of spring flowering phenology related to climate variability and change with pollen dynamics and asthma hospitalization risk. We further provide evidence associating changes in spring flowering phenology with pollen season length, although this evidence is not from the same location. Our findings regarding increased risk of asthma hospitalization are consistent with our previous work,^52^ which found that changes in SOS were associated with increased prevalence of hay fever in the contiguous United States. However, this previous study was based on self-reported prevalence of hay fever during a 12-month period. As such, the exact date of symptom onset was unknown, which precluded us from establishing temporality between exposure and outcome. The use of asthma hospitalization in the current study overcomes the limitation regarding unknown date of symptom onset, given that the hospitalization likely followed asthma exacerbation within a short interval.

### Strengths and Limitations

There are several strengths of this study, including our relatively large sample size (29 257 springtime asthma hospitalizations) that spanned 12 years. Our use of relative change in the timing of SOS rather than the actual date minimizes the uncertainties in NDVI data that may vary by location, depending on the vegetation type and spatial coverage. Likewise, we only considered deciduous vegetation coverage, which allowed us to exclude changes in greenness associated with agricultural activity or other vegetation types, which do not contribute to pollen exposure during spring.

Our study has several limitations as well. For example, there may not be a complete temporal alignment between exposure (changes in phenology date) and spring hospitalization records, given that the latter include all hospitalizations between March and May. It should be noted that during the 12-year study period, 2010 had the highest number of county-years during which spring onset was 10 days early. While we adjusted for extreme heat events and air pollution levels, it remains unclear how additional unmeasured confounders associated with this particular year may explain some of the findings we observed. It is worth noting that additional sensitivity analysis excluding 2010 data did not change our overall findings. We were unable to account for the severity of the influenza season, given that we did not have county-level influenza data dating back to 2001. While our hospitalization data are from Maryland, we had to rely on pollen data from eastern Canada to evaluate whether changes in timing of spring onset were in fact associated with pollen dynamics, including start and length of pollen season as well as timing and intensity of peak pollen concentration. This was out of necessity because Maryland does not have speciated pollen data. Because of privacy concerns, hospitalization data were available to us at the county level. Future studies need to consider finer geographic scales for analysis, given that there can be heterogeneity in the timing of spring onset within a county, particularly if it includes urban centers as well as suburban and rural locations. Such studies should include larger and more diverse geographic regions and longer periods to further identify underlying population vulnerability as well as geographic variability in risk. Similarly, future studies should also include additional health outcomes that are not associated with pollen exposure as negative controls to ensure that other temporal factors are not responsible for the observed association between changes in spring phenology and asthma hospitalization.

## Conclusions

To our knowledge, this study has provided the first empirical evidence that changes in the timing of spring onset related to ongoing climate variability and change are associated with increased risk of asthma hospitalization. Our results serve as a wake-up call to patients with asthma as well as public health professionals regarding the need to anticipate and adapt to the ongoing changes in the timing of the spring allergy season. Our findings also highlight the opportunity for leveraging readily available satellite observations to inform location-specific, personalized early warning systems to minimize asthma burden.

## Notes

Supplement. **eFigure.** Timing of Spring Onset in the 24 Counties in Maryland During 2001 to 2012

## References

[1] Ebi KL, Balbus JM, Luber G, Human Health: Impacts, Risks, and Adaptation in the United States: Fourth National Climate Assessment. Vol II U.S. Global Change Research Program; 2018:572-603.

[2] US Global Change Research Program. The impacts of climate change on human health in the United States: a scientific assessment. Crimmins A, Balabus J, Gamble CB, , eds. Published 2016 Accessed June 5, 2020. https://health2016.globalchange.gov/

[3] Hamilton JA, El Kayal W, Hart AT, Runcie DE, Arango-Velez A, Cooke JE The joint influence of photoperiod and temperature during growth cessation and development of dormancy in white spruce (Picea glauca). Tree Physiol. 2016;36(11):1432-1448. doi:10.1093/treephys/tpw061 27449791

[4] Cook BI, Wolkovich EM, Parmesan C Divergent responses to spring and winter warming drive community level flowering trends. Proc Natl Acad Sci U S A. 2012;109(23):9000-9005. doi:10.1073/pnas.1118364109 22615406 PMC3384199

[5] Bock A, Sparks TH, Estrella N, Changes in first flowering dates and flowering duration of 232 plant species on the island of Guernsey. Glob Chang Biol. 2014;20(11):3508-3519. doi:10.1111/gcb.12579 24639048

[6] Menzel A, Sparks TH, Estrella N, European phenological response to climate change matches the warming pattern. Glob Change Biol. 2006;12(10):1969-1976. doi:10.1111/j.1365-2486.2006.01193.x

[7] Amano T, Smithers RJ, Sparks TH, Sutherland WJ A 250-year index of first flowering dates and its response to temperature changes. Proc Biol Sci. 2010;277(1693):2451-2457. doi:10.1098/rspb.2010.0291 20375052 PMC2894925

[8] Menzel A Trends in phenological phases in Europe between 1951 and 1996. Int J Biometeorol. 2000;44(2):76-81. doi:10.1007/s004840000054 10993561

[9] Parmesan C, Yohe G A globally coherent fingerprint of climate change impacts across natural systems. Nature. 2003;421(6918):37-42. doi:10.1038/nature01286 12511946

[10] Wolkovich EM, Cook BI, Allen JM, Warming experiments underpredict plant phenological responses to climate change. Nature. 2012;485(7399):494-497. doi:10.1038/nature11014 22622576

[11] Mazer SJ, Travers SE, Cook BI, Flowering date of taxonomic families predicts phenological sensitivity to temperature: implications for forecasting the effects of climate change on unstudied taxa. Am J Bot. 2013;100(7):1381-1397. doi:10.3732/ajb.1200455 23752756

[12] Bolmgren K, Vanhoenacker D, Miller-Rushing AJ One man, 73 years, and 25 species: evaluating phenological responses using a lifelong study of first flowering dates. Int J Biometeorol. 2013;57(3):367-375. doi:10.1007/s00484-012-0560-8 22744801

[13] Emberlin J, Mullins J, Corden J, The trend to earlier birch pollen seasons in the UK: a biotic response to changes in weather conditions? Grana. 1997;36(1):29-33. doi:10.1080/00173139709362586

[14] Emberlin J, Smith M, Close R, Adams-Groom B Changes in the pollen seasons of the early flowering trees Alnus spp. and Corylus spp. in Worcester, United Kingdom, 1996-2005. Int J Biometeorol. 2007;51(3):181-191. doi:10.1007/s00484-006-0059-2 17024396

[15] Li X, Zhou Y, Asrar GR, Meng L Characterizing spatiotemporal dynamics in phenology of urban ecosystems based on Landsat data. Sci Total Environ. 2017;605-606:721-734. doi:10.1016/j.scitotenv.2017.06.245 28675882

[16] Cai H, Zhang S, Yang X Forest dynamics and their phenological response to climate warming in the Khingan Mountains, northeastern China. Int J Environ Res Public Health. 2012;9(11):3943-3953. doi:10.3390/ijerph9113943 23202825 PMC3524606

[17] Cong N, Wang T, Nan H, Changes in satellite-derived spring vegetation green-up date and its linkage to climate in China from 1982 to 2010: a multimethod analysis. Glob Chang Biol. 2013;19(3):881-891. doi:10.1111/gcb.12077 23504844

[18] Walther GR, Post E, Convey P, Ecological responses to recent climate change. Nature. 2002;416(6879):389-395. doi:10.1038/416389a 11919621

[19] Donnelly A, Yu R The rise of phenology with climate change: an evaluation of *IJB* publications. Int J Biometeorol. 2017;61(suppl 1):29-50. doi:10.1007/s00484-017-1371-8 28527153

[20] Ahas R, Aasa A The effects of climate change on the phenology of selected Estonian plant, bird and fish populations. Int J Biometeorol. 2006;51(1):17-26. doi:10.1007/s00484-006-0041-z 16738902

[21] Galán C, García-Mozo H, Vázquez L, Ruiz L, de la Guardia CD, Trigo MM Heat requirement for the onset of the Olea europaea L. pollen season in several sites in Andalusia and the effect of the expected future climate change. Int J Biometeorol. 2005;49(3):184-188. doi:10.1007/s00484-004-0223-5 15645246

[22] Estrella N, Menzel A, Krämer U, Behrendt H Integration of flowering dates in phenology and pollen counts in aerobiology: analysis of their spatial and temporal coherence in Germany (1992-1999). Int J Biometeorol. 2006;51(1):49-59. doi:10.1007/s00484-006-0038-7 16832654

[23] Li X, Zhou Y, Meng L, Asrar G, Sapkota A, Coates F Characterizing the relationship between satellite phenology and pollen season: a case study of birch. Remote Sens Environ. 2019;222:267-274. doi:10.1016/j.rse.2018.12.036

[24] D’Amato G, Cecchi L Effects of climate change on environmental factors in respiratory allergic diseases. Clin Exp Allergy. 2008;38(8):1264-1274. doi:10.1111/j.1365-2222.2008.03033.x 18537982

[25] Ziska LH, Makra L, Harry SK, Temperature-related changes in airborne allergenic pollen abundance and seasonality across the northern hemisphere: a retrospective data analysis. Lancet Planet Health. 2019;3(3):e124-e131. doi:10.1016/S2542-5196(19)30015-4 30904111

[26] Emberlin J, Detandt M, Gehrig R, Jaeger S, Nolard N, Rantio-Lehtimäki A Responses in the start of Betula (birch) pollen seasons to recent changes in spring temperatures across Europe. Int J Biometeorol. 2002;46(4):159-170. doi:10.1007/s00484-002-0139-x 12242471

[27] Osborne NJ, Alcock I, Wheeler BW, Pollen exposure and hospitalization due to asthma exacerbations: daily time series in a European city. Int J Biometeorol. 2017;61(10):1837-1848. doi:10.1007/s00484-017-1369-2 28500390 PMC5643363

[28] Sun X, Waller A, Yeatts KB, Thie L Pollen concentration and asthma exacerbations in Wake County, North Carolina, 2006-2012. Sci Total Environ. 2016;544:185-191. doi:10.1016/j.scitotenv.2015.11.100 26657364

[29] Ito K, Weinberger KR, Robinson GS, The associations between daily spring pollen counts, over-the-counter allergy medication sales, and asthma syndrome emergency department visits in New York City, 2002-2012. Environ Health. 2015;14:71. doi:10.1186/s12940-015-0057-0 26310854 PMC4549916

[30] Jariwala SP, Kurada S, Moday H, Association between tree pollen counts and asthma ED visits in a high-density urban center. J Asthma. 2011;48(5):442-448. doi:10.3109/02770903.2011.567427 21453203

[31] Erbas B, Jazayeri M, Lambert KA, Outdoor pollen is a trigger of child and adolescent asthma emergency department presentations: a systematic review and meta-analysis. Allergy. 2018;73(8):1632-1641. doi:10.1111/all.13407 29331087

[32] Taylor PE, Jacobson KW, House JM, Glovsky MM Links between pollen, atopy and the asthma epidemic. Int Arch Allergy Immunol. 2007;144(2):162-170. doi:10.1159/000103230 17536216

[33] Gilmour MI, Jaakkola MS, London SJ, Nel AE, Rogers CA How exposure to environmental tobacco smoke, outdoor air pollutants, and increased pollen burdens influences the incidence of asthma. Environ Health Perspect. 2006;114(4):627-633. doi:10.1289/ehp.8380 16581557 PMC1440792

[34] Blackwell DL, Lucas JW, Clarke TC Summary health statistics for U.S. adults: National Health Interview Survey, 2012. Vital Health Stat 10. 2014;(260):1-161. 24819891

[35] Bloom B, Cohen RA, Freeman G Summary health statistics for U.S. Children: National Health Interview Survey, 2011. Vital Health Stat 10. 2012;(254):1-88. 25116332

[36] US Centers for Disease Control and Prevention Asthma in the US. Published May 2011. Accessed June 5, 2020. https://www.cdc.gov/vitalsigns/asthma/index.html

[37] Cherry DK, Hing E, Woodwell DA, Rechtsteiner EA National Ambulatory Medical Care Survey: 2006 summary. Natl Health Stat Report. 2008;(3):1-39. 18972720

[38] DeFrances CJ, Podgornik MN 2004 National Hospital Discharge Survey. Adv Data. 2006;2008(371):1-19. 16703980

[39] National Heart, Lung, and Blood Institute Morbidity and Mortality: 2009 Chart Book on Cardiovascular, Lung, and Blood Diseases. National Institutes of Health; 2009.

[40] Zhang Y, Bielory L, Mi Z, Cai T, Robock A, Georgopoulos P Allergenic pollen season variations in the past two decades under changing climate in the United States. Glob Chang Biol. 2015;21(4):1581-1589. doi:10.1111/gcb.12755 25266307 PMC4356643

[41] Ziello C, Sparks TH, Estrella N, Changes to airborne pollen counts across Europe. PLoS One. 2012;7(4):e34076. doi:10.1371/journal.pone.0034076 22514618 PMC3325983

[42] Ariano R, Canonica GW, Passalacqua G Possible role of climate changes in variations in pollen seasons and allergic sensitizations during 27 years. Ann Allergy Asthma Immunol. 2010;104(3):215-222. doi:10.1016/j.anai.2009.12.005 20377111

[43] Soneja S, Jiang C, Fisher J, Upperman CR, Mitchell C, Sapkota A Exposure to extreme heat and precipitation events associated with increased risk of hospitalization for asthma in Maryland, U.S.A. Environ Health. 2016;15:57. doi:10.1186/s12940-016-0142-z 27117324 PMC4847234

[44] USGS Remote sensing phenology. Accessed June 9, 2020. https://www.usgs.gov/land-resources/eros/phenology/data-tools

[45] Reed BC, Brown JF, VanderZee D, Loveland TR, Merchant JW, Ohlen DO Measuring phenological variability from satellite imagery. J Veg Sci. 1994;5(5):703-714. doi:10.2307/3235884

[46] Brown J, Howard D, Wylie B, Frieze A, Ji L, Gracke C Application-ready expedited MODIS data for operational land surface monitoring of vegetation condition. Remote Sens. 2015;7(12):16226-16240. doi:10.3390/rs71215825

[47] Fry J, Xian G, Jin S, Completion of the 2006 National Land Cover Database for the conterminous United States. Photogramm Eng Remote Sensing. 2011;77:858-863. Accessed June 5, 2020. http://digital.ipcprintservices.com/publication/index.php?m=&l=1&i=78634&p=8&ver=html5

[48] Garcia-Mozo H, Oteros J, Galan C Phenological changes in olive (Ola europaea L.) reproductive cycle in southern Spain due to climate change. Ann Agric Environ Med. 2015;22(3):421-428. doi:10.5604/12321966.1167706 26403107

[49] Darrow LA, Hess J, Rogers CA, Tolbert PE, Klein M, Sarnat SE Ambient pollen concentrations and emergency department visits for asthma and wheeze. J Allergy Clin Immunol. 2012;130(3):630-638.e4. doi:10.1016/j.jaci.2012.06.020 22840851 PMC3432157

[50] Xie ZJ, Guan K, Yin J Advances in the clinical and mechanism research of pollen induced seasonal allergic asthma. Am J Clin Exp Immunol. 2019;8(1):1-8. 30899604 PMC6420698

[51] Stinson KA, Albertine JM, Hancock LM, Seidler TG, Rogers CA Northern ragweed ecotypes flower earlier and longer in response to elevated CO_2_: what are you sneezing at? Oecologia. 2016;182(2):587-594. doi:10.1007/s00442-016-3670-x 27318697 PMC5021721

[52] Sapkota A, Murtugudde R, Curriero FC, Upperman CR, Ziska L, Jiang C Associations between alteration in plant phenology and hay fever prevalence among US adults: implication for changing climate. PLoS One. 2019;14(3):e0212010. doi:10.1371/journal.pone.0212010 30921361 PMC6438449

