# Supplementary material for: Association Between Changes in Timing of Spring Onset and Asthma Hospitalization in Maryland
Source: JAMA Netw Open. 2020 Jul 6;3(7):e207551. doi: 10.1001/jamanetworkopen.2020.7551 (PMC7339136; doi:10.1001/jamanetworkopen.2020.7551)
Supplement: Supplement. — eFigure. Timing of Spring Onset in the 24 Counties in Maryland During 2001 to 2012 [file jamanetwopen-3-e207551-s001.pdf]

Supplementary Online Content

Sapkota A, Dong Y, Li L, et al. Association between changes in timing of spring onset and asthma hospitalization in Maryland. *JAMA Netw Open*. 2020;3(7):e207551. doi:10.1001/jamanetworkopen.2020.7551

eFigure. Timing of Spring Onset in the 24 Counties in Maryland During 2001 to 2012

This supplementary material has been provided by the authors to give readers additional information about their work.

© 2020 Sapkota A et al. *JAMA Network Open.*

eFigure. Timing of Spring Onset in the 24 Counties in Maryland During 2001 to 2012

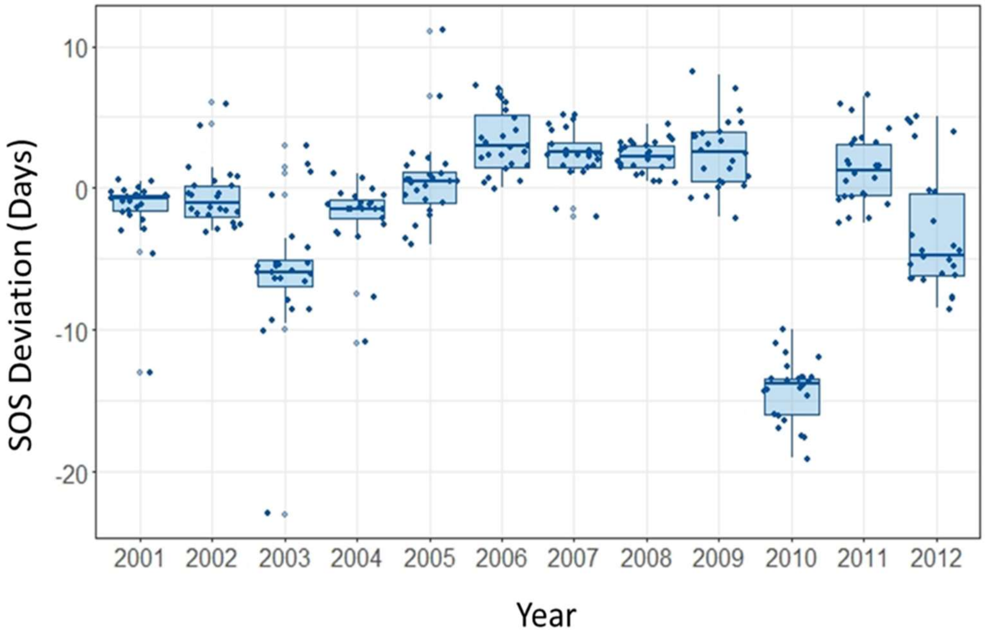

© 2020 Sapkota A et al. *JAMA Network Open.*
